# Extracting the Jugular Venous Pulse from Anterior Neck Contact Photoplethysmography

**DOI:** 10.1038/s41598-020-60317-7

**Published:** 2020-02-26

**Authors:** Irene García-López, Esther Rodriguez-Villegas

**Affiliations:** Department of Electrical and Electronic Engineering, Imperial College London, London, SW7 2AZ United Kingdom

**Keywords:** Prognostic markers, Cardiology, Cardiovascular diseases, Diagnosis, Medical imaging

## Abstract

The jugular venous pulse (JVP) is the reference physiological signal used to detect right atrial and central venous pressure (CVP) abnormalities in cardio-vascular diseases (CVDs) diagnosis. Invasive central venous line catheterization has always been the gold standard method to extract it reliably. However, due to all the risks it entails, novel non-invasive approaches, exploiting distance cameras and lasers, have recently arisen to measure the JVP at the external and internal jugular veins. These remote options however, constraint patients to very specific body positions in front of the imaging system, making it inadequate for long term monitoring. In this study, we demonstrate, for the first time, that reflectance photoplethysmography (PPG) can be an alternative for extracting the JVP from the anterior jugular veins, in a contact manner. Neck JVP-PPG signals were recorded from 20 healthy participants, together with reference ECG and arterial finger PPG signals for validation. B-mode ultrasound imaging of the internal jugular vein also proved the validity of the proposed method. The results show that is possible to identify the characteristic *a, c, v* pressure waves in the novel signals, and confirm their cardiac-cycle timings in consistency with established cardiac physiology. Wavelet coherence values (close to 1 and phase shifts of ±180°) corroborated that neck contact JVP-PPG pulses were negatively correlated with arterial finger PPG. Average JVP waveforms for each subject showed typical JVP pulses contours except for the singularity of an unknown "u" wave occurring after the *c* wave, in half of the cohort. This work is of great significance for the future of CVDs diagnosis, as it has the potential to reduce the risks associated with conventional catheterization and enable continuous non-invasive point-of-care monitoring of CVP, without restricting patients to limited postures.

## Introduction

Cardiovascular diseases (CVDs) are listed as the principal cause of mortality worldwide by the World Health Organization (WHO). Accounting for 17.9 million deaths annually, they represent 31% of global deceases^1^. In Europe, CVDs claim 3.9 million deaths per year with an estimated total cost of 210 billion euros to the European Union^2^. In the United States, 92.1 million people are currently living with some variety of CVD^3^. CVDs result in an impaired blood supply to the different organs of the body. They include vascular disorders, involving blocked or damaged blood vessels (e.g., coronary, peripheral or cerebrovascular arterial disease); and cardiac disorders, resulting from heart contractility dysfunction (e.g. cardiomyopathy, heart failure, cardiac dysrhythmias).

Non-invasive diagnostic and monitoring methods for CVDs include the assessment of cardiac electrical activity with electrocardiography (ECG), examination of blood arterial pulsations with photoplethysmography (PPG) or ballistocardiograhy, and scrutiny of the vasculature’s function by measuring arterial blood pressure (BP). Most of these physiological signals provide information about cardiac pump efficiency and blood delivery function. However, a significant number of CVDs affecting the right side of the heart cannot be diagnosed with these, since they result in central venous pressure (CVP) abnormal values that cannot be inferred from the arterial pulse. In these cases, the jugular venous pulse (JVP), is the standard physiological signal commonly measured for diagnosis^4^. Examples of CVDs that can be diagnosed by identifying JVP abnormalities are: hypovolaemia, tricuspid stenosis and regurgitation, constrictive pericarditis, atrio-ventricular block, atrial fibrillation, or cardiac tamponade^5^.

**Figure 1:**
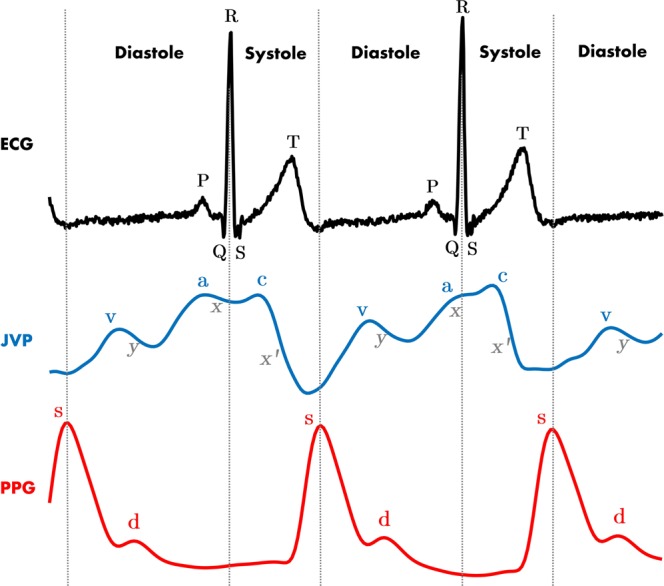
ECG, JVP and PPG signals traces. Dashed vertical lines separate the two phases of the cardiac cycle: systole and diastole. Each physiological signal is annotated with the corresponding significant waves. For ECG: *P* corresponds to atrial depolarization, *QRS* complex to ventricular depolarization and *T* to ventricular repolarization. For JVP: the *a,c,v* ascents are annotated in blue and the *x, x’* and *y* descents in gray. For PPG: *s* indicates the systolic peak and *d* the diastolic peak.

The JVP is considered as a manometer for right atrial pressure and CVP, because the superior vena cava extends all the way from the entrance of the right atrium up to the internal jugular vein at the neck^6^. Due to the quasi-absence of bifurcations between the first and the latter, the pressure at the right atrium is transmitted following a straight route to the internal jugular vein. Moreover, during diastole, while the tricuspid valve is open, the right ventricle is directly connected in series with the right atrium, superior vena cava and ultimately with the jugular vein. This results in differentiating characteristics of the JVP signal when there is an abnormal behaviour of the right ventricle too. The typical JVP tracing is presented in Fig. 1, together with the typical ECG and PPG signals for cardiac time reference purposes.

The JVP waveform is formed by three ascents (*a, c* and *v* waves) and three descents (*x, x’*and *y*), which respectively represent the different events of the cardiac cycle in terms of pressure variations. The (*a*) wave, occurring right after the *P* peak of the ECG, represents atrial contraction and is followed by the (*x*) descent that indicates relaxation of the atrium and closure of the tricuspid valve. Subsequently, after the *QRS* complex of the ECG, the (*c*) wave shows right ventricular contraction as a result of the bulging of the tricuspid valve during ejection, and it is followed by the drop in pressure (*x’*). Finally, after ventricular repolarization (*T* peak of ECG), the (*v*) wave reflects the maximum pressure attained from blood filling of the right atrium before the tricuspid valve opens again. This last event results in the fall of pressure during rapid ventricular filling, represented by the (*y*) wave; and the whole cycle starts again^7^. The typical arterial PPG waveform onset happens approximately at the start of the diastolic phase, but it can occur earlier or later in time, specific to the person, as it depends on the pulse transit time to the periphery.

The most broadly used technique to measure the jugular venous pulse (JVP) is central venous line cathetherization. This consists in surgically introducing a catheter into the right internal jugular vein, all the way to the right atrium and sometimes even advancing it down the pulmonary artery^8^. This invasive method is not routinely performed in primary care due to all the risks it entails, but instead, it is only used in acute CVD patients at the ICU^9^. Some undesirable complications that justify this decision include: pneumothorax, carotid artery puncture, pulmonary infarction, arryhthmias or catheter infection^10^.

The alternative non-invasive methods that eliminate the risks of such an invasive technique, look for JVP waveform abnormalities by indirectly observing the internal pressure of the heart at the more accessible site of the external neck. This can be done, for example, by simple visual examination of the jugular vein blood column height in the triangle formed by the sternocletomastoid muscles and the clavicle^11^. However, this option requires trained personnel and is usually very subjective, making its accuracy questionable. Alternatively, a study also showed the feasibility of extracting the JVP using ultrasound (US) B-mode of the jugular vein cross-sectional area^12^. Although this method showed potential, a stable probe-skin contact is crucial and US equipment remains very expensive.

Non-invasive remote optical techniques that try to counteract the disadvantages of the current state-of-the-art have been explored recently. Some initial efforts visualising the neck area with a camera system equipped with a near-IR light, were able to identify neck pulsations in the triangle of the sternocleidomastoid muscles underlying the right jugular vein^13^. However, this proof of concept was unable to distinguish whether the pulsations were of carotid or jugular origin, to prove their hypothesis. A different approach, based on a video imaging photoplethysmographic system, demonstrated that, with the subject still in supine position it was feasible to extract the JVP waveform from the neck^14^. Another recent study^15^, showed that camera-based skin micro-motion vibrocardiography (cVCGI) with subjects in recumbent-to-supine position could sense skin-displacement caused by jugular vein pulsations. Similarly, Lam Po Tang *et al*.^16^ non-contact alternative was based on using a comodity camera and a subpixel image registration algorithm to identify the same jugular skin displacements.

However, despite the promising proposed methods in the literature, all these video remote diagnostic systems constraint the patient to a specific body position facing the camera in front of them. This severely restricts the patients from moving or changing position during monitoring, making it unsuitable for longer term signal acquisition. In addition, in the clinical setting, they all require training of hospital personnel to ensure correct set up and equipment calibration.

This paper shows, for the first time, that it is possible to extract the JVP from the anterior jugular veins by using contact photoplethysmography (PPG). The advantage of this is that PPG is an easy and cheap physiological sensing modality, and thus, a system developed using this could potentially overcome the shortcomings of all other existing techniques. The fundamentals insights provided by this study are intended to set the foundations for the future design of a small, light and user-friendly wearable sensor, which would be able to record continuously for long periods of time, without constraining the position of the patient in bed. This technique thus, has the potential to be a breakthrough for physiological monitoring of CVDs, mostly in outpatient clinics, domestic environments, and low resourced settings.

## Results

### Anterior neck veins imaging

**Figure 2:**
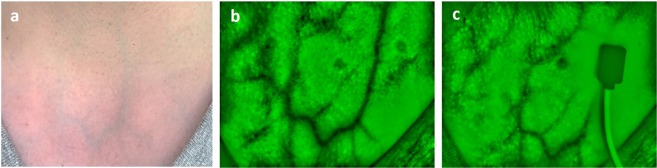
Anterior view of neck veins (subject 12). (**a**) Image of the anterior lower neck. Veins are already visible to the naked eye. (**b**) Vein camera image of anterior jugular veins and jugular venous arch. (**c**) Reflectance PPG sensor placed on top of the left anterior jugular vein for recordings.

 Figure 2 shows the anterior view of the venous system at the lower neck, visible to the naked eye (a) and by means of the IR camera system (b-c). As it can be observed in the leftmost panel 2(a), veins are very evident already in the selected participant with very pale thin skin. It is not always that obvious for participants with darker skin tones, which is why the IR imaging system was used to confirm the underlying venous anatomy. With the IR camera it was possible to visualize with a low resolution (and hence in some subjects better than in others) the pair of anterior jugular veins (AJVs) descending parallel to the midline of the neck and connecting at the jugular venous arch at the inferior part, as shown in Fig. 2(b). The rightmost panel 2(c), demonstrates a possible longitudinal positioning of the reflectance PPG sensor on top of the left AJV, that enables extraction of the desired neck JVP-PPG signal.

**Figure 3:**
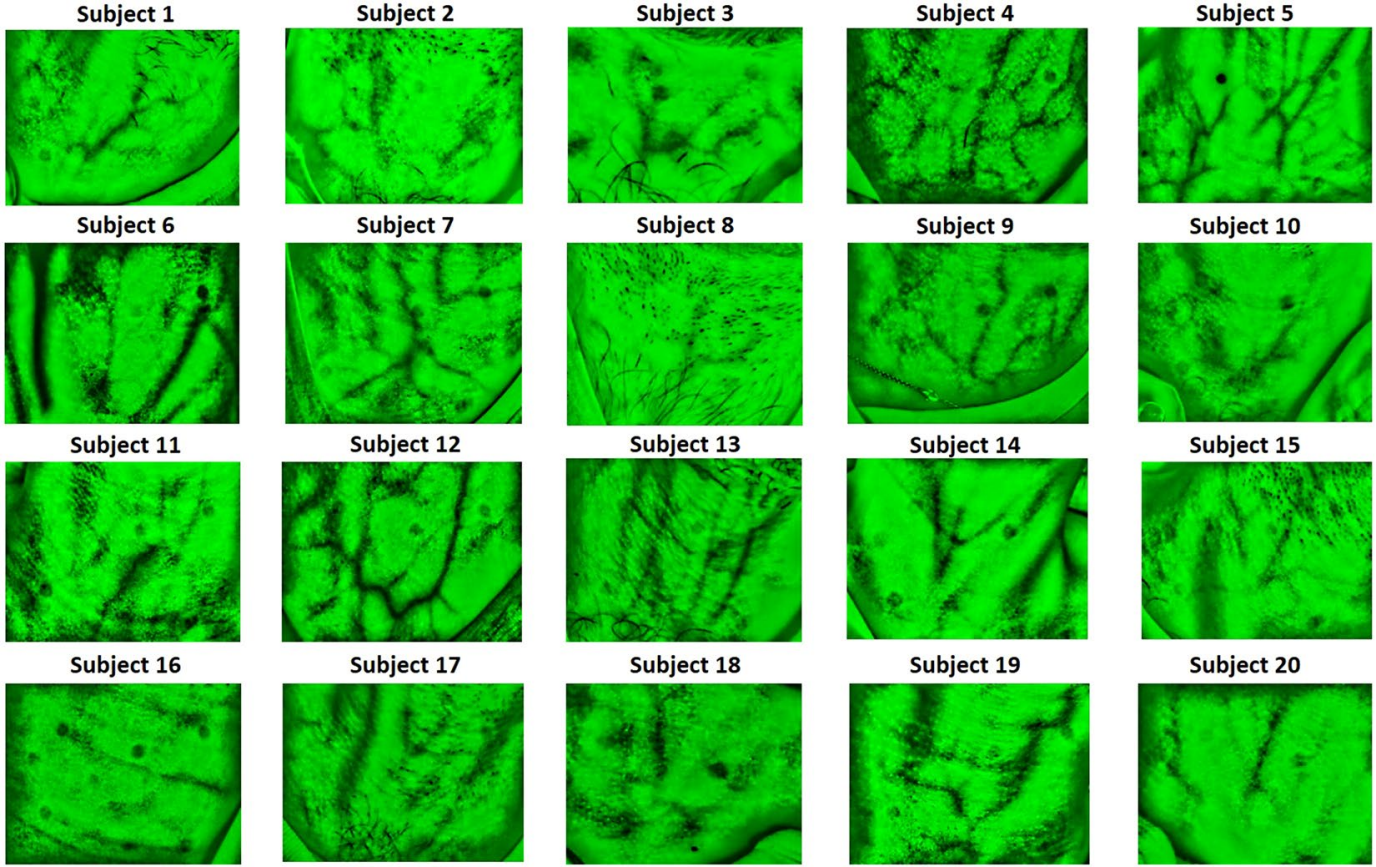
Anterior neck veins vasculature imaging for all participants (n = 20).

Vein vasculature imaging, at the anterior neck, varies significantly among subjects as it can be observed in Fig. 3. Differences in anatomy and fat content in the neck, affect the identification of veins that are located more or less superficially. For some subjects (4, 5, 7, 9, 12, 13, 19); broad venous ramifications are clearly noticeable as intertwined black curvilinear lines. AJVs connecting into the jugular venous arch can be identified in most of the images. For other subjects (2, 8, 10, 16, 18), the expected venous tree is hardly observable, but instead, less sharp grey lines highlight some thinner isolated veins.

### Ultrasound internal jugular vein measurements validation

**Figure 4:**
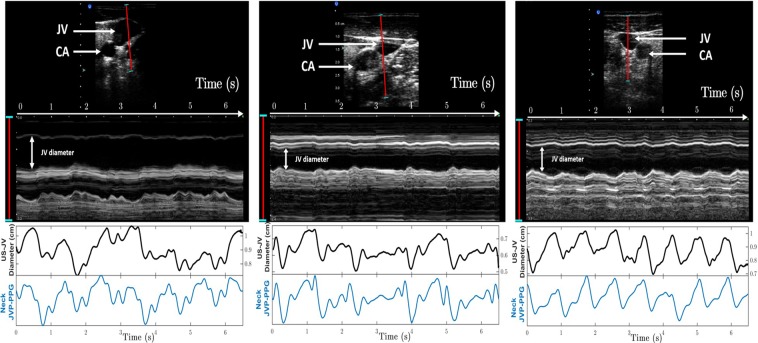
Validation of the proposed neck JVP-PPG signals with reference ultrasound (US-JVP) for three different subjects. Top panels show the B-mode image of the carotid artery (CA) and jugular vein (JV) in the transverse plane together with the 2D cross-section profile along the red (3.2 cm) segment over time. The middle panel displays the US-derived JVP signal, i.e. the JV cross-sectional diameter variations over time, after image post-processing of the US 2D topography for the specified time interval. The bottom panel presents the corresponding neck JVP-PPG signal recorded in parallel over the 6.5s period of time, that perfectly follows the reference US-JVP morphology.

 Figure 4 shows the comparison between the ultrasound (US) derived jugular venous pulse and our proposed neck JVP-PPG signals, for three different subjects. The top panel shows the US B-mode image of the carotid artery (CA) and jugular vein (JV). In red, the segment of 3.2 cm in length, indicates along which line the cross-section of the jugular vein was measured at each time instant for a duration of 6.5 seconds. Underneath, the resulting 2D topography graph shows the jugular venous walls and surrounding tissues displacement in the transverse plane over the recording period. The middle panel shows the extracted US jugular venous pulse (US-JVP) after segmenting uniquely the JV cavity area profile. This US-JVP corresponds to the variation in cross-sectional jugular diameter (in cm) over time. When comparing this US reference waveform morphology to our neck JVP-PPG signals, shown in the bottom panel, it can be seen that they match perfectly in terms of shape and timings. In both signals, US-JVP and neck JVP-PPG, the typical (*a,c,v*) JVP waves can be identified. This validates our proposed neck contact PPG technique for extracting the JVP from the anterior neck.

### Wavelet coherence between neck JVP and finger PPG signals

The raw neck and finger PPG signals measured for all subjects are shown in Figure 1 of the Supplementary Information. As it can be observed, the two PPG signals are not in phase, but instead the neck JVP pulses seem to lead the arterial finger PPG pulses. This is already consistent with cardiac cycle physiology, since the JVP *a* and *c* waves mark the right atrial and ventricular contractions, that give rise to the arterial PPG pulse observed *a posteriori* at the peripheral parts of the body.

**Figure 5:**
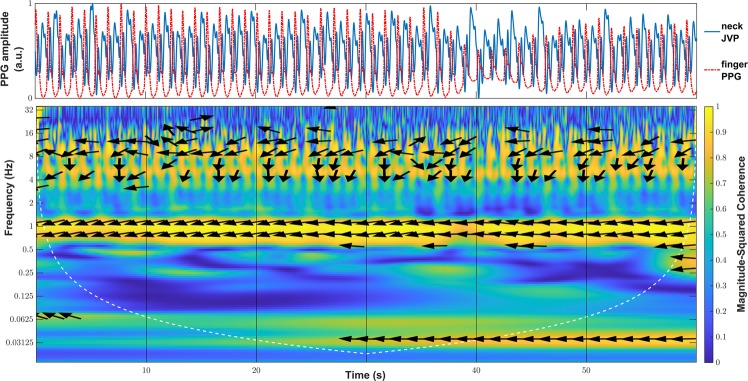
Wavelet coherence plot for neck inverted JVP and arterial finger PPG signals. Arrows represent the phase of the wavelet cross-spectrum at magnitude-squared coherence values greater than 0.8.

Indeed, an interesting property of the JVP identified by Amelard *et al*.^14^ was that it is strongly negatively correlated with the arterial PPG pulse. In order to verify whether our recorded signals were also inverted, the magnitude-squared wavelet coherence was computed, as a measure of the correlation in the time-frequency plane between neck inverted JVP and reference finger PPG signals. The coherence plot of these, is shown for one of the subjects in Fig. 5. The phase values of the wavelet cross-spectrum are represented by arrows, the orientation of which indicates the relative lag between the neck JVP and finger PPG pulses. Phase arrows are only displayed for coherence values larger than a manually set threshold of 0.8, therefore showing strong correlation.

At the cardiac frequency of 1Hz there is a concentration of large wavelet coherence values almost equal to 1, between the neck inverted JVP and the reference finger PPG signals. In addition, along this band, for the whole duration of the recording, it can also be observed that the phase arrows are pointing in the antiphase direction (±180°). This confirms the inversion of the proposed neck contact JVP with respect to the arterial pulse, and hence the strong negative correlation.

### Neck JVP signals annotation and characteristic waves location

**Figure 6:**
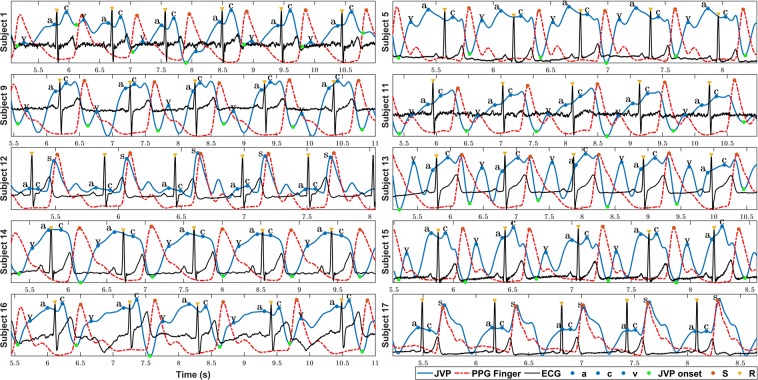
Manual *a, c, v* waves annotation of neck JVP signals with respect to ECG and PPG reference signals for 10 subjects. **S**: finger arterial PPG systolic peak. **R**: QRS complex peak of the ECG. **a**,**c**,**v**: characteristic JVP waves.

 Figure 6 shows the neck JVP signals manually annotated (blue dots), together with the ECG (yellow diamonds), and PPG (red dots) reference signals, for 10 subjects. The three characteristic JVP *a, c* and *v* waves were identified in all subjects, except for subject 20 for which the *a* wave was not recognized. The green dots point the onsets of the JVP pulses used to calculate the *v-v* interval distances. These were needed to normalize the locations of the different waves and peaks at each cycle, and to calculate the differences between them. Note that for subjects 12 and 17, the neck JVP signals appear as a mix of the expected venous JVP and the arterial PPG pulse. Indeed the latter part is so prominent that it seems to overlap with the finger PPG ground-truth signal. Due to this, it was not possible to accurately define the location of the *v* wave and, therefore, the *v* wave was annotated. The onsets of the JVP pulses could not be defined and, as a consequence, no time differences calculations were considered in these two cases (12&17).

**Figure 7:**
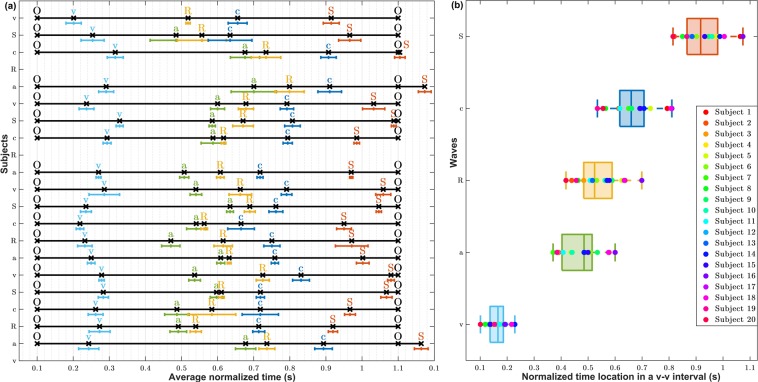
Location of the annotated *a, c, v, R* and *S* peaks within a JVP cycle. (**a**) Average normalized locations of the most characteristic JVP, ECG, PPG waves over a *v-v* cycle for each subject (n = 20). (**b**) Boxplots summarizing the normalized time locations for each *v, a, c, R* and *S* waves for all participants (n = 20).

For the rest of the subjects, all the characteristic JVP waves were succesfully annotated. To verify the consistency of this annotation accross subjects, Fig. 7(a) shows the normalized location of each *a, c, v, R* and *S* waves within the *O-O* interval averaged over 5 JVP cycles for each subject. Standard deviation error bars were also specified. As it can be observed, overall for all subjects, the order of waves in the normalized *v-v* interval is: *v, a, R, c, S*. This corresponds to the same cardiac cycle timings recognized in the literature, as presented in Fig. 1. This confirms the validity of the signals, but also shows that the JVP waveform is susceptible to change its morphology over time, as the standard deviations appear quite variable depending on the subject. Also for some subjects (1,16 and 18) it can be noticed that the systolic peak of finger PPG exceeds the normalized *O-O* interval boundary, as the *R* peak occurs later in time for these subjects.

Figure 7(b)presents a summary of the order and time location distributions of each peak for all subjects, in the form of boxplots. These give an idea of the range in which each significant peak is expected to occur within the *v-v* interval, between the lower and upper adjacent values. Based on these results, the *v* wave occurrence is in the range of [0.101–0.228] of the normalized *v-v* interval of a JVP pulse; the *a* wave in [0.370–0.600], the *R* peak in [0.418–0.699], the *c* wave in [0.534–0.810], and the *S* peak in [0.815–1.074]. As it can be observed, all distributions show similar dispersion, apart from the *v* wave which is slightly smaller. Also, they all appear symmetric, except for the *a* wave which is slightly skewed.

### Timings between JVP *a, c, v* waves and ECG and PPG peaks

**Figure 8:**
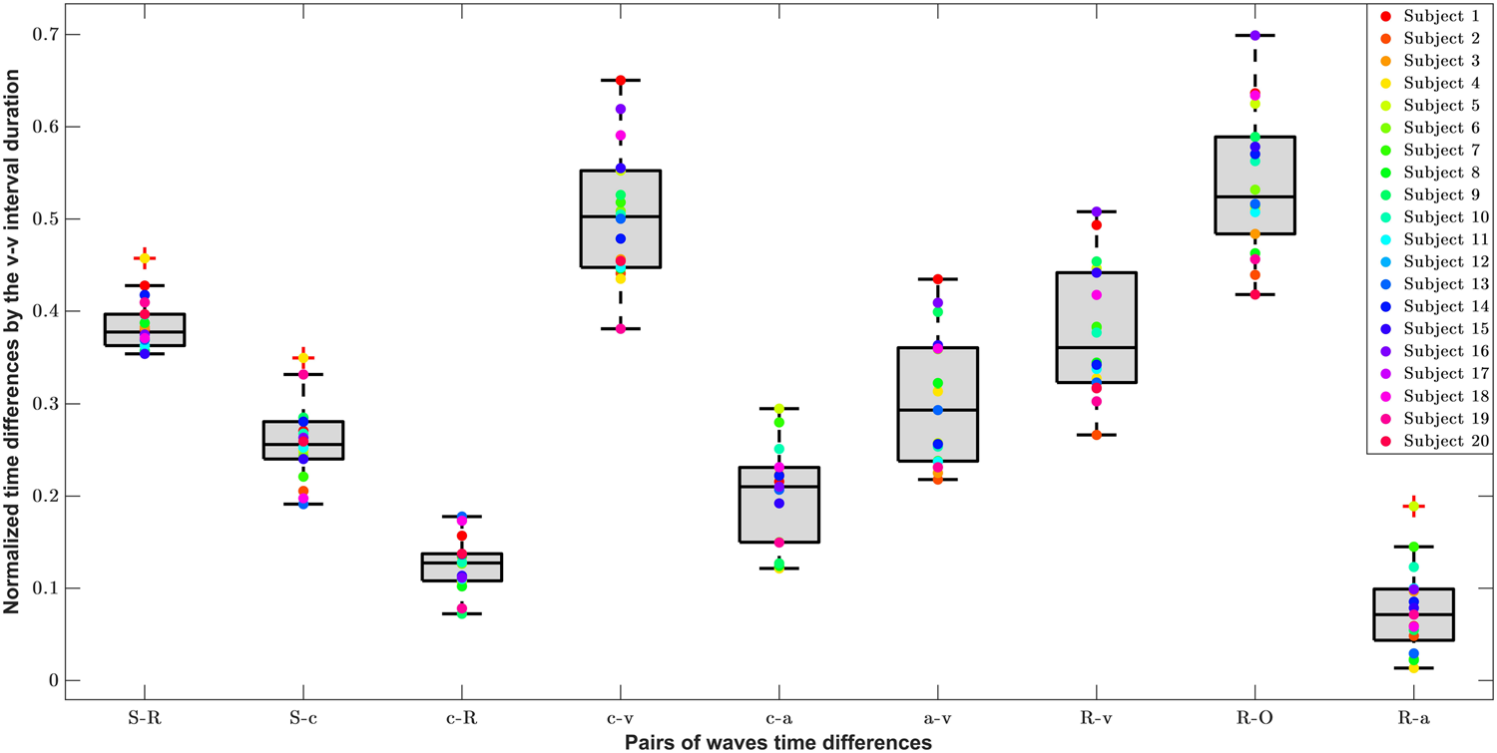
Boxplots showing the distributions of average time differences between characteristic peaks and waves from neck JVP, finger PPG and ECG (n = 20). **S**: arterial finger PPG systolic peak. **R**: QRS complex peak of the ECG. **a**,**c**,**v**: characteristic JVP waves. **O**: onset of the JVP pulse.

The relative timings between the main (*a,c,v*) waves and the *R* and *S* peaks, were calculated to compare with the durations extracted by Lam Po Tang *et al*.^16^ in the work quantifying the JVP from skin displacements. Even if that study only considered the *v-R* and *R-c* time differences, which correspond to the *R-O* and *c-R* respectively in this work, the following were also investigated: *S-R*, *S-c*, *c-v*, *c-a*, *a-v*, *R-v*, *R-a*. Average and standard deviations values of the normalized time differences are presented for each subject in Table 1 (Supplementary Information). Figure 8 summarizes the information for all subjects by means of boxplots.

As it can be observed in Fig. 8, the largest time differences, normalized by the *v-v* interval duration, correspond to the *R-O* (JVP onset to EGC peak) and *c-v* waves pairs, with median values of 0.52 and 0.50, accordingly. The smallest time difference found is for the *R-a* pair, with a median value of 0.071, as ventricular depolarisation (*R*) directly follows atrial contraction (*a*) in the cardiac cycle time sequence. Similarly, *c-R* time variations also show a small median value of 0.13.

Focusing on the JVP waves differences, it can be noticed that in increasing order, *c-a* has the shortest distance (med = 0.21), followed by *a-v* (med = 0.29), and *c-v* has the largest duration (med = 0.50). This is consistent with the literature, which validates the manual annotation of these novel neck JVP-PPG signals and shows the potential they have to be clinically used for biomarkers extraction.

The distance between the PPG systolic peak and the ECG peak (*S-R*), i.e. the pulse transit time (PTT), showed a median normalized value of 0.38. This was larger than the distance between the same PPG peak and the JVP *c* wave (*S-c*) (med = 0.25), which is in accordance with the cardiac cycle too, as *c* wave always occurs after the QRS complex of the ECG.

*R-v* and *R-O* refer to the same distance, but the *R-v* measure proposed in this work is calculated from the *v* peak to *R* peak, whereas *R-O* is the equivalent to *v-R* measured by Lam Po Tang *et al*.^16^. It was observed that they shared more or less similar ranges in the *v-v* normalized interval. In this work, the normalized *c-R* interval ranged from 72.3 ± 13.4 (subject 9) to 177.7 ± 15.4 (subject 13); whereas in their work it ranged from 139.6 ± 6.1 to 248.3 ± 10.5. For the normalized *R-O*, values ranged between 418 ± 5.9 and 699 ± 40.7; whilst their *v-R* interval spanned from 360.7 ± 8.0 and 547.2 ± 20.4.

Overall, the normalized time differences showed symmetric distributions except for the *c-a* waves which appeared slightly skewed. The dispersion of the data varied for the various pairs. For *S-R*, *S-c* and *c-R*, the dispersion was very small, with interquartile range (IQR) values of 0.034, 0.40 and 0.29 respectively; for *R-a* (IQR = 0.055) and *c-a* (IQR = 0.081) was medium; and for the rest larger, with IQR around 0.1. The variance for each subject time difference also varied depending on the pair of waves. The average standard deviation for each *c-R* and *S-c* point was around 0.010; for *S-R*, *R-a*, *c-a*, *c-v*, was around 0.013 and for *a-v*, *r-v* and *R-O* of 0.02.

### Average JVP waveforms for all subjects

**Figure 9:**
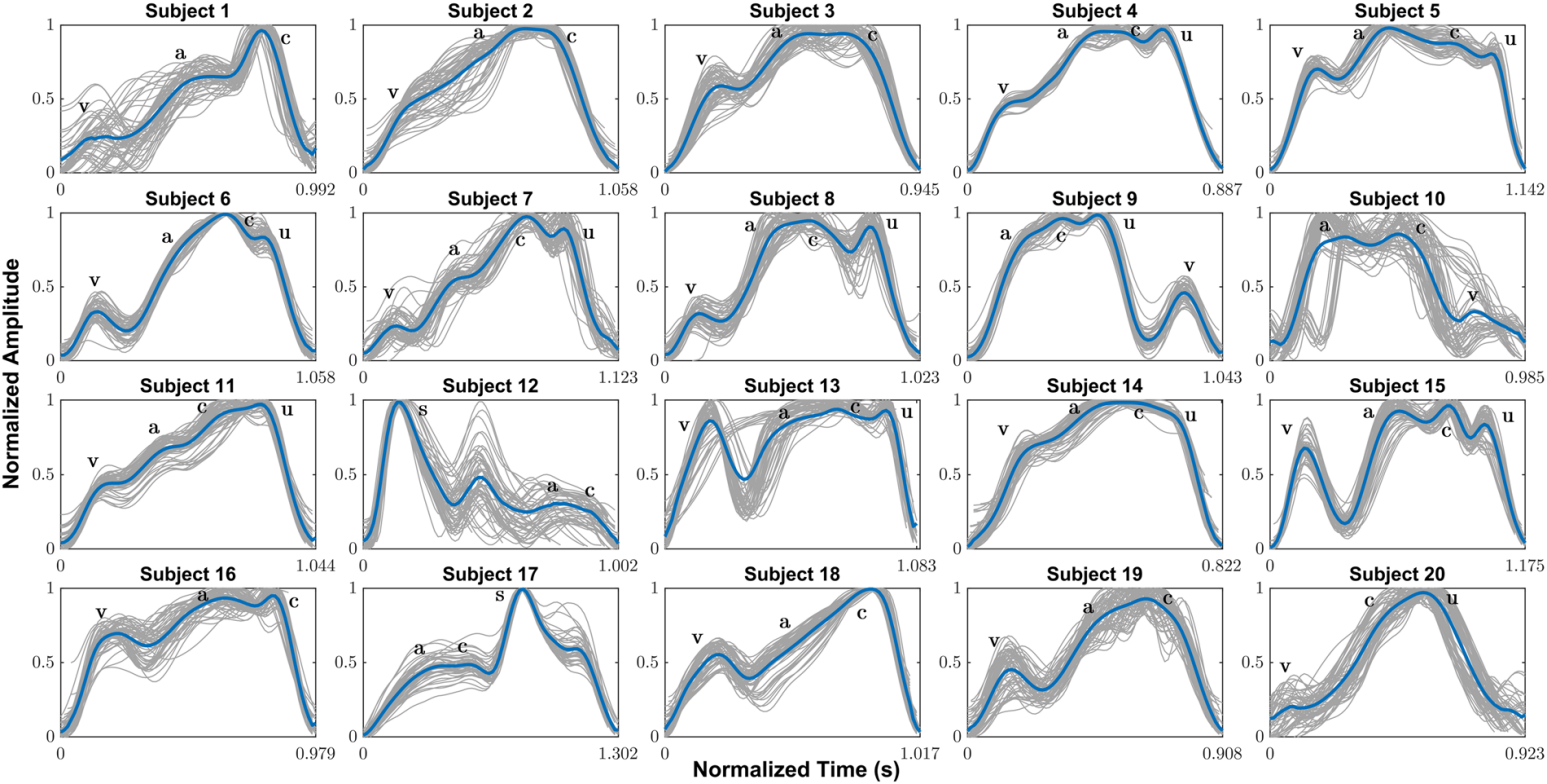
Average JVP waveforms manually annotated for all participants (n = 20). **a,c,v**: characteristic JVP waves as described in the literature. **u**: unknown extra peak specific to the proposed method. **S**: arterial systolic peak overlapping with JVP waveform.

 Figure 9 shows the mean neck JVP waveforms extracted for the 20 participants. The pulse shapes in blue, resulted from the averaging of all the individual JVP pulses present in the whole length of the recording (60 s), plotted in grey. Despite the fact that the JVP morphology is subject to change from one cardiac cycle to another (as in subjects 1, 10 or 13), in the majority of the cases the individual pulses appeared very similar to each other, facilitating the computation of an accurate average estimate. As a result, the characteristic *a, c, v* waves were marked, based on the manual annotation performed on each subject’s recording with respect to reference ECG and PPG signals.

Different types of average JVP pulse shapes were visualized among different subjects. In all the cases, the *v, a* and *c* waves could be clearly identified visually, except for subject 20, for whom the *a* wave did not show enough prominence to be annotated. Indeed, in the majority of the signals (e.g. subjects 1, 6, 7, 8, 9, 10, 13, 15, 18), the *v* wave appears significantly isolated in time, with a prominent *y* descent, compared to the *a* and *c* greater amplitude waves. For half of the participants in the study (subjects 4, 5, 6, 7, 8, 11, 13, 14, 15, 20), a total of 4 distinctive waves were noticed. The first three were marked as the JVP characteristic peaks and the last one was denoted as “*u*”, standing for unknown. For subjects 12 and 17, the average pulse shape reflects the superposition of the venous JVP *a* and *c* waves, and the arterial PPG pulse with the distinctive *S* peak.

Moreover, some average JVP waveforms show a more triangular shape (e.g. subjects 2, 3, 4, 11, 14, 16), with the *v* wave closer in time to the *a* and *c* waves, collapsing the *y* descent slope which appears very flat or non-existent. This happened independently of the presence of the 4th additional “*u*” wave.

## Discussion

In this paper, the feasibility of extracting the JVP waveform from the anterior neck, using a reflectance PPG sensor, was demonstrated. Neck JVP-PPG signals were recorded from a total of 20 participants in supine position. Since it is the first time that the JVP is sensed with a contact PPG sensor from the anterior neck, a series of validation metrics were presented to further support the hypothesis and conclusions of this work, as well as to assess the similarities and differences with other methods.

Although it was possible to obtain the JVP signal in the majority of the subjects, JVP-PPG signals were not equally accessible for all participants at once. For some, an exhaustive exploration of the anterior neck area with the PPG sensor was necessary to find the most suitable sensing location, while in others it was straightforward. A possible reason is the differences in anatomy. Indeed, depending on the subject, veins are likely to be located less superficially in the tissue or occluded by more fat, which would avoid the IR light to penetrate very deep subcutaneously. This was confirmed with the IR camera system that showed how the vein vasculature of the anterior neck appeared visibly different from one subject to another. However, it is worth pointing out that a basic IR imaging low cost system was chosen for the proof of concept in this study as first approach, and might not have revealed the complete venous vasculature precisely.

In other cases, despite relocating the sensor and confirming with the camera that there was a vein underneath, it was still not obvious how to obtain the JVP signals. A clear example was subject 12, for whom vein imaging was one of the most clear ones among all subjects, but all the sensor locations explored resulted in recordings with a mix of the venous JVP and arterial PPG pulses. Indeed the PPG arterial mixed part of the neck JVP-PPG signal was almost perfectly matching in time with the reference PPG pulses by a small difference of 0.05s. A possible hypothesis is that the subject was very athletic, meaning that its stroke volume and cardiac output would be higher. The increase in the force of ventricular contraction allows an increased amount of blood to be ejected, resulting in an arterial pulse of higher amplitude at the neck, being sensed straight away following the *a* and *c* waves of the JVP pulse. Another possibility, however, might have been, that the light is passing through superposed veins and arterioles or at different skin depths, this causing the two signals to be sensed simultaneously, as a mix, in a delayed manner.

On another extreme, in some cases, such as for subject 19, it was not possible to identify the neck arterial PPG pulse anywhere in the neck. It was only possible to extract the JVP (even when exploring the suprasternal notch area that is typically optimal for neck arterial PPG recording, as previously demonstrated^17^). This could be due to the fact that the venous JVP pulse is stronger than the arterial one, even in regions that are supposed to be more irrigated by arterioles. Probably, a reduced cardiac output in this subject was the cause of an increase in CVP.

In this study it was demonstrated, for the first time, that novel neck JVP-PPG signals can be extracted from the anterior neck veins using a contact PPG reflective sensor. Ultrasound reference measurements of the jugular vein in the cross-sectional transverse plane, validated our proposed method.

The computation of the wavelet coherence corroborated the inversion property of the JVP waveform with respect to arterial PPG in concordance with negative correlation observed by Amelard *et al*.^14^. Indeed, this verifies that the proposed neck JVP signal really represents the atrial and ventricular contractions of the right heart (*a-c* waves) that typically occur before the arterial pulse (PPG) is transmitted towards the periphery (finger).

The three characteristic JVP waves *a, c, v* were identified in the majority of the signals, except for subject 20 for whom the *a* wave was not recognized. The order of the peaks in the normalized *v-v* interval appeared to be *v, a, R, c, S*, consistent with cardiac physiology. This demonstrates that, the novel contact neck JVP-PPG signals recorded in this study, morphologically present all the conventional features of the well-known JVP and, have the potential to be exploited for CVDs clinical diagnosis. However, the location of each JVP wave was observed to be slightly variable. This suggests that the JVP waveform is predisposed to change its timing morphology from one cardiac cycle to another, as already suggested by Lam Po Tang *et al*.^16^. However, we additionally defined the normality ranges of the different waves within the JVP cycle for the proposed technique, to be able to reliably detect abnormalities in JVP *a, c, v* timings that appear outside of the established bounds.

When analysing the time differences between the JVP *a, c, v* waves and the ECG and PPG peaks, it was noticed how the dispersion of the boxplots varied from one pair of waves to another. The reduced dispersion in *S-R*, *S-c*, *c-R* distributions could be explained by the fact that ECG and PPG peaks, are well known and accurately detected, and they represent, together with the *c* wave, the same physiological process of systolic contraction. So, it could be expected that the time variations within the cardiac cycle between these dependent processes would not vary much among subjects in a healthy population sample. On the opposite, larger variability of time differences between other JVP waves pairs (*c-v*, *a-v*, *R-v* and *R-O*), could be explained by the dissimilarities in anterior veins vasculature and cardiac system anatomy, unique to each individual.

The comparison with previous work by Lam Po Tang *et al*.^16^, of the *c-R* and *R-O* time intervals, showed comparable range of values but not exactly the same. The small inexactitude could be due to the smaller cohort of subjects (6 in total) that they used to calculate these differences, compared to the 18 participants we employed. But the close similarity, validates our neck contact JVP-PPG signals as a commensurable method for non-invasive JVP monitoring.

For the first time, average JVP waveforms were computed for each subject. A wider variety of JVP pulse morphologies were observed than in previous works measuring the JVP remotely at the external or internal jugular vein^14–16^. The different pulse shape classes could be a specific trait of the sensing location of the anterior neck, or simply different morphologies were not revealed in previous studies because averaging of the obtained JVP waveforms was not investigated.

The most distinctive feature of our contact neck JVP-PPG signals, is the presence of an extra 4th wave, of unknown origin, denoted as “*u*” and located after the *c* wave. The recurrent occurrence of this, in half of the cohort, led us to discard the idea that its origin was the result of some random artifact. After measuring some neck arterial PPG signals from the suprasternal notch and verifying the close superposition with the reference finger PPG, we abandoned the idea that it was the result of the mixing of the venous JVP and arterial PPG pulses (as in subjects 12 & 17). We therefore hypothesize that the origin of this “*u*” wave is the delayed arrival of a pressure wave reflected through the intricate venous tree. Indeed, as discussed in the Methods, the anterior jugular veins can either connect directly to the subclavian vein or indirectly via the external jugular vein. The latter will introduce an extra bifurcation in the direct path between the RA and the measurement site at the anterior neck. Additionally, if we consider other bifurcations in the different veins (thyroid, anterior or internal jugular) that connect and intertwingle at the anterior neck, this will increase the likelihood of reflections sites. Therefore, singularities in different people’s anatomy and the confluence of different venous blood columns might explain the appearance of new waves in the neck contact JVP contour and the diversity in morphology.

These findings set for the first time the key attributes of the novel neck contact JVP-PPG signals and add extra value to the state-of-the-art of JVP recording techniques. Our proposed method is an easy to use, low cost solution for measuring JVP non-invasively from the anterior neck. It benefits from not requiring expensive equipment or infrastructure, as others solutions do. Unlike previous studies, that extract the JVP from the external and internal jugular veins, this work explores for the first time the original sensing location of the anterior jugular veins. Another advantage is that no expert personnel is required for JVP exploration due to the simplicity of moving the sensor around the anterior neck for signal acquisition. These findings will have a great impact in reducing the risks associated to central venous line catheterization, and therefore, are very promising for the future of CVDs clinical diagnosis. In spite of this, this technique suffers from some minor limitations. It is not always easy to locate the JVP in all subjects equally, due to the variability in veins anatomy, fat content, and pressure in the venous system. Therefore, future research should focus on tackling some of the open questions that remain unanswered. Is there an optimal sensor location and specific conditions to measure this neck contact JVP-PPG waveform in a more standardized manner? What is the origin of the additional unknown “*u*” wave? Is it relevant for JVP monitoring and diagnosis? In future studies, a deeper exploration of the unique intricate venous anatomy should help understand the differences in JVP waveforms morphologies. Ultimately, a clinical validation study, to assess the neck contact PPG modality against invasive catheterization should be carried out to evaluate the viability of implementing our alternative in continuous CVDs monitoring.

## Conclusions

This work proposes, for the first time in literature, the use of reflectance contact PPG on the anterior neck, as a non-invasive, low cost alternative to sense JVP and obtain physiological parameters of relevance for CVDs. Data acquisition from a total of 20 participants provided a snapshot of the pressure variations occurring at the right atrium of the heart, as reference ultrasound measurements of the internal jugular vein validated. To demonstrate the hypothesis (i.e. that the JVP could be observed through PPG), as well as the feasibility of the new proposed method, the characteristic *a, c, v* waves of the neck JVP-PPG signals were manually annotated with the help of some reference ECG and finger arterial PPG signals. Calculation of time differences between significant features confirmed the validity of the novel JVP signals, their annotation, and the consistency with previous methods in the literature. Wavelet coherence values proved that neck JVP-PPG signals were also inversely correlated with respect to reference arterial finger PPG. In addition, the extracted average neck JVP waveforms highlighted some singularities of the presented technique. Despite the fact that all distinctive *a, c, v* waves could be identified, some pulse shapes showed a more triangular contour than typical, as a result of the reduced prominence of the y descent following the *v* wave. An additional “*u*” wave, of unknown origin, appeared unexpectedly right after the *c* wave in half of the JVPs in the cohort.

These findings are of great significance for the future design of low cost, wearable PPG-based sensors to continuously monitor changes in central venous pressure. Patients could easily wear the sensor, with the only condition being having to comfortably lie down during measurements; as opposed to having to resort to either invasive and/or non-invasive but more restrictive and costly methods. Thus, this will aid in both, the efficiency in diagnosis of CVDs, as well as their management; whilst also eliminating some of the risks of invasive alternatives.

## Methods

### Experimental protocol

Signals were recorded from 20 subjects (15 males and 5 females), with average age 27 ± 4 years old, height of 1.75 ± 0.09 m and weight of 74.2 ± 13.1 kg. The study was approved by the Local Ethics Committee of Imperial College London (ICREC ref.: 18IC4358), and written informed consent was obtained from all subjects. Informed consent was likely provided from participants whose images were displayed in this paper, for both study participation and publication of identifying information/images in an online open-access publication. All experiments in this work were performed in accordance with the Declaration of Helsinki. The subjects were asked to be in a lying down position, since it was hypothesized (and confirmed) that JVP pulsations could not be properly sensed if participants were seated^15^. This is due to the fact that when the body is lying down, the venous return does not have to counteract the effect of gravity to pump blood back to the heart from the lower extremities, and blood is pumped more easily to the head. In fact, neck venous pressure is elevated, enhancing the pulsatility of the JVP pulse at the neck.

One reflectance pulse oximeter sensor (8000R, Nonin) coupled to an OEM processing module (Xpod, Nonin), was located on the anterior area of the neck. A transmission finger pulse oximeter (Onyx II 9560, Nonin) with Bluetooth connectivity, was located on the index finger of the left hand, and used as the ground truth. In order to validate and identify more precisely the JVP characteristic (*a,c,v*) waves and their corresponding timings, 2-lead electrocardiography (ECG) sensors from a polysomnography system (SOMNOscreenplus, SOMNOmedics), were also used as reference. A PPG sensor connected to the polysomnography system and placed on the index finger of the right hand, was used to synchronize with the two Nonin PPG sensors.

In order to further confirm the relative position of the reflectance neck PPG sensor, with respect to the neck veins, an IR vein camera (Infrared Vein Finder, Z-imaging) connected via USB to a computer, was placed 30 cm above the participant’s head. The mounted imaging system, that was used to visualize the underlying venous system of the anterior neck, consisted of a 1080P IR sensitive lens surrounded by IR LEDs (800–1000 nm) to illuminate the tissue.

**Figure 10:**
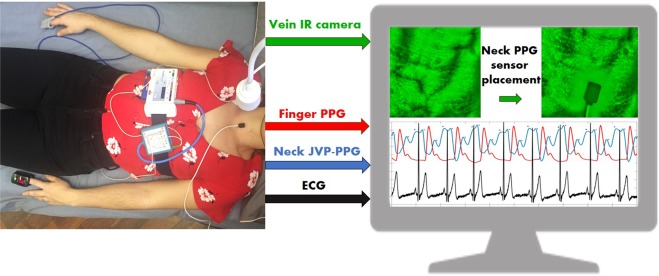
Experimental setup. Participant lying down with two transmission pulse oximeters on each hand and one reflectance PPG sensor on the neck. ECG signals were simultaneously recorded using a portable polysomnography system (SOMNOmedics). The vein imaging camera was placed 30 cm on top of the participant’s neck to visualize the neck vasculature. Online vein imaging, neck JVP-PPG signals and ground truth finger PPG, were observable in real-time on the screen of the computer. ECG and PPG signals acquired in parallel with the polysomnography system were synchronized and processed after the data acquisition session was finished.

 Figure 10 shows the experimental setup, with one of the subjects lying down in supine position on a bed with all the attached sensors and imaging system. The IR vein camera online acquisition and Nonin PPG sensors recordings could be visualized in real-time on the screen to verify the venous anatomy of the neck area. Signals were acquired for 60 seconds duration for each subject. After data acquisition, ECG recordings were imported into the computer for processing. Recordings were synchronized in time by finding the maximum correlation between both fingers PPG sensors’ signals.

A diagnostic ultrasound (US) system (Sonix RP, Ultrasonix) coupled with a linear transducer probe (14-5 MHz) was also used to obtain transverse B-mode images and videos of the internal jugular vein in order to validate our PPG-based jugular measurements. Neck JVP-PPG recordings were acquired in parallel with the US probe placed on the right side of the neck for 6.5 s. Using the 3.2 cm cross-section measurement tool of the US system, the jugular vein wall distension across the imaged internal jugular vein was measured in the transverse plane over time. The output 2D topographic graph (of 6.5 s duration) was further processed to segment the jugular vein cavity area and calculate the cross-sectional diameter at each instant of time. For that, all the pixels inside the segmented jugular walls were summed and scaled in terms of distance (cm). The resulting variations in diameter of the jugular cross-section represent the reference ultrasound JVP waveform for comparison with our neck JVP-PPG signals.

### Sensing location

**Figure 11:**
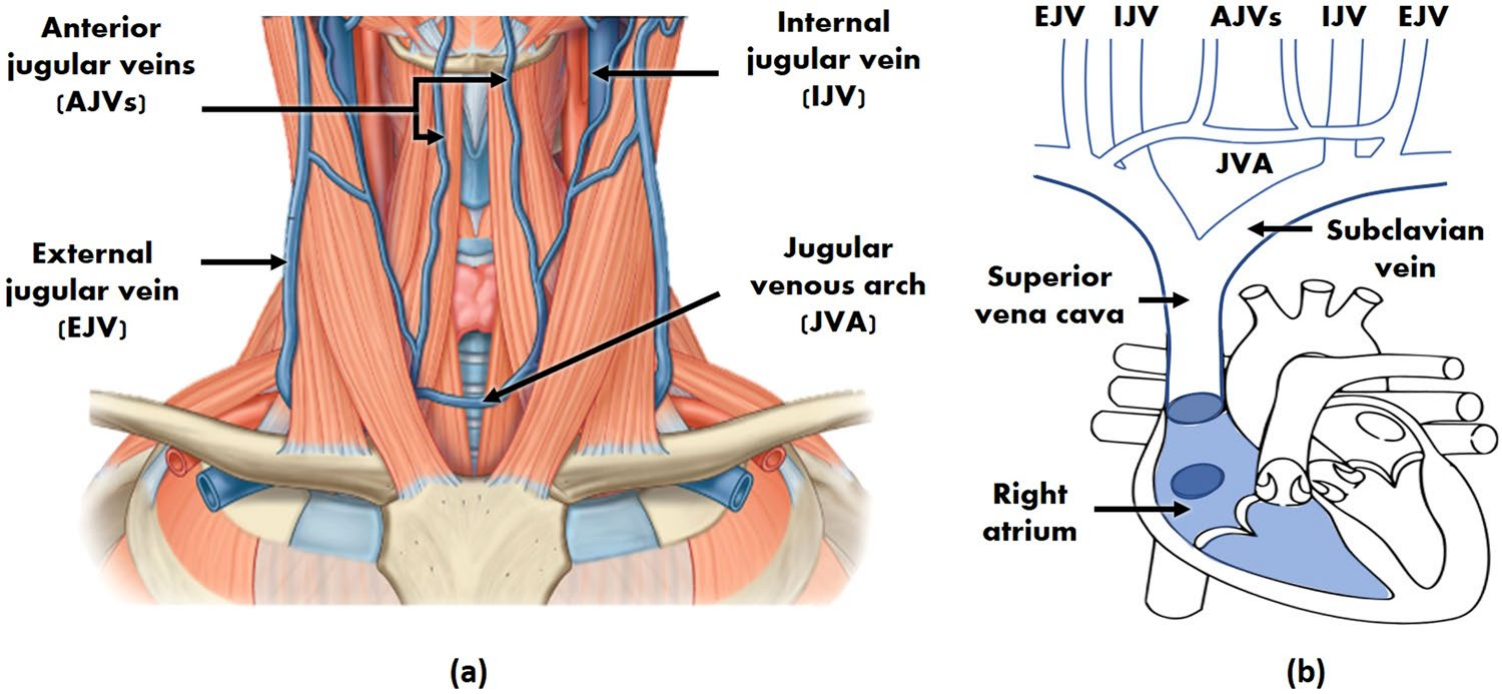
Neck venous system anatomy. (**a**) Superficial veins schematic of the inferior anterior region of the neck (adapted from^18^). (**b**) Schematic of the venous tree connecting the external (EJV), internal (IJV) and anterior (AJVs) jugular veins to the right atrium of the heart.

In order to extract the JVP from the anterior neck, the contact reflectance PPG sensor was placed at the middle inferior region. As it can be observed in Fig. 11(a), this area is highly perfused by the anterior jugular veins (AJVs) that combine into the jugular venous arch (JVA)^18,19^. The JVA drains into the subclavian vein (or occasionally into the external jugular vein (EJV)), which directly joins the superior vena cava further down, and ultimately the right atrium of the heart, as shown in Fig. 11(b). This venous configuration connecting the neck jugular veins in almost a straight path with the right side of the heart, allows RA pressure changes to be easily transmitted to the AJVs and JVA, in the form of the JVP.

But the JVP is only noticeable in this area of interest, when the participant’s body is in recumbent or supine positions^15^. For this reason, experiments were carried out with participants lying down. Blood then concentrates more in the central venous compartment as a result of being less influenced by the gravitational pull, and hence, the JVP can be better observed in the neck venous system.

Another key feature that makes the frontal neck suitable for reflectance contact PPG sensing is the absence of thick tissues preventing the penetration of light. Neither the sternocleidomastoid nor the platysma superficial muscle cover the central lower area along the midline. On the contrary, the AJVs appear exposed in front of the infrahyoid muscles, and accessible superficially as they pierce the investing fascia^18^. Moreover, the skin thickness of the anterior neck is narrower than for the anterior-lateral regions^20^. In addition, the inferior part, is the thinnest compared to the middle and superior neck regions. These anatomical properties facilitate the light to easily reach the subcutaneous plexus.

### Neck JVP and finger PPG signals correlation analysis

In order to measure the correlation in the time-frequency plane between the neck JVP and finger ground-truth PPG signals the magnitude-squared wavelet coherence was used. It was computed using the analytic Morlet wavelet over logarithmic scales, with a default value of 12 voices per octave. For two signals *x* and *y*, it is defined as:

1 $$WCoherenc{e}_{xy}^{2}(f)=\frac{|{C}_{xy}(f){|}^{2}}{{C}_{xx}(f){C}_{yy}(f)}=\frac{|s({C}_{x}^{\ast }(a,b){C}_{y}(a,b)){|}^{2}}{s(|({C}_{y}(a,b){|}^{2})s(|({C}_{y}(a,b){|}^{2})}$$

where, *C*_*xy*_(*f*) denotes the wavelet cross-spectrum between *x* and *y*, and *C*_*xx*_(*f*) and *C*_*yy*_(*f*) the wavelet auto-spectra of *x* and *y* respectively. *C*_*x*_(*a*, *b*) and *C*_*y*_(*a*, *b*) refer to the continuous wavelet transforms of *x* and *y* at scales *a* and positions *b*. *s(·)* represents the smoothing operator in time and scale, and ^*^ the complex conjugate. The magnitude-squared wavelet coherence values range from 0 to 1, i.e. from low to high correlation. The phase of the wavelet cross-spectrum values were also extracted to inspect the relative lag between the neck JVP and finger PPG signals.

### Neck JVP waveforms annotations and averaging

For each subject, signals segments of 5 seconds duration were selected. JVP signals were manually annotated by marking the characteristic (*a, c, v*) waves, as well as the onset of each JVP pulse corresponding to the trough before each *v* wave. The main peaks of the ECG (*R*) and arterial finger PPG (*S*) were also identified for reference.

Average time differences between ECG, PPG peaks and JVP waves were calculated for 5-cycles. Each JVP cycle (i.e. *v-v* interval) was defined as the duration between two subsequent onsets (*O*), and was used to normalize the distances between waves. This was done similarly to Lam Po Tang *et al*.^16^, for the sake of comparison.

Using the whole duration of the recording (60 s), average JVP pulse shapes were obtained for each subject. For that, the automatic algorithm previously presented in^17^, was applied to perform segmentation, alignment and averaging with a quality correction stage, of JVP pulses. The average waveforms were annotated, after being normalized in amplitude and in time (by the average pulse duration), for inter-subjects comparison.

## Supplementary information

Supplementary Information.

## References

[1] World Health Organization (WHO). Cardiovascular diseases (CVDs), http://www.who.int/en/news-room/fact-sheets/detail/cardiovascular-diseases-(cvds) (2017).

[2] Wilkins, E. *et al*. European cardiovascular disease statistics 2017. Tech. Rep., European Heart Network (2017).

[3] Benjamin EJ (2018). Heart disease and stroke statistics-2018 update: a report from the American Heart Association. Circulation.

[4] McGee, S. R. Inspection of the neck veins. In *Evidence-based physical diagnosis*, chap. 32 (Saunders, 2001).

[5] Garg N, Garg N (2000). Jugular venous pulse: an appraisal. Journal, Indian Academy of Clinical Medicine.

[6] Constant J (2000). Using internal jugular pulsations as a manometer for right atrial pressure measurements. Cardiology.

[7] Ranganathan, N., Sivaciyan, V. & Saksena, F. B. Jugular venous pulse. In *The Art and Science of Cardiac Physical Examination*, 67–111 (Springer, 2006).

[8] Smith RN, Nolan JP (2013). Central venous catheters. Bmj.

[9] Tibby S, Murdoch I (2003). Monitoring cardiac function in intensive care. Archives of Disease in Childhood.

[10] Mathews, R. & Brown, D. L. Invasive hemodynamic monitoring in the cardiac intensive care unit. In *Cardiac intensive care*, chap. 2nd edn. **45**, 558–569 (Saunders, 2010).

[11] Applefeld, M. M. The jugular venous pressure and pulse contour. In *Clinical methods: The history, physical, and laboratory examinations* 3rd edn. chap. 19, 107 (Butterworth-Heinemann, 1990)

[12] Sisini F (2015). An ultrasonographic technique to assess the jugular venous pulse: a proof of concept. Ultrasound in medicine & biology.

[13] Dang, T. T., Huynh, C., Dinh, A. & Tran, K. Recognizing area of pulsations on the neck via video camera systems. In *International Conference on Advanced Technologies for Communications (ATC)*, 139–144 (IEEE, 2015).

[14] Amelard R (2017). Non-contact hemodynamic imaging reveals the jugular venous pulse waveform. Scientific Reports.

[15] Moço, A., Hamelmann, P. & Haan, G. The importance of posture and skin-site selection on remote measurements of neck pulsations: An ultrasonographic study. In *40th Annual International Conference of the IEEE Engineering in Medicine and Biology Society (EMBC)*, 5918–5921 (IEEE, 2018). 10.1109/EMBC.2018.8513651 30441683

[16] LamPoTang EJ (2018). Non-contact quantification of jugular venous pulse waveforms from skin displacements. Scientific reports.

[17] García-López, I., Imtiaz, S. A. & Rodriguez-Villegas, E. Rodriguez-Villegas, E. Characterization study of neck photoplethysmography. In *40th Annual International Conference of the IEEE Engineering in Medicine and Biology Society (EMBC)*, 4355–4358 (IEEE, 2018). 10.1109/EMBC.2018.8513247 30441318

[18] Drake, R., Vogl, A. W. & Mitchell, A. W. *Gray’s anatomy for students*. 3rd edn. (Elsevier Health Sciences, 2015).

[19] Dalip, D., Iwanaga, J., Loukas, M., Oskouian, R. J. & Tubbs, R. S. Review of the variations of the superficial veins of the neck. *Cureus* **10** (2018). 10.7759/cureus.2826 PMC6101467 30131919

[20] Chopra K (2015). A comprehensive examination of topographic thickness of skin in the human face. Aesthetic Surgery Journal.

