# Supplementary material for: Extracting the Jugular Venous Pulse from Anterior Neck Contact Photoplethysmography
Source: Sci Rep. 2020 Feb 26;10:3466. doi: 10.1038/s41598-020-60317-7 (PMC7044195; doi:10.1038/s41598-020-60317-7)
Supplement: Supplementary file 1 — Supplementary Information. [file 41598_2020_60317_MOESM1_ESM.pdf]

Supplementary information: Extracting the Jugular Venous Pulse from Anterior Neck Contact Photoplethysmography

Irene García-López[1,*] and Esther Rodriguez-Villegas[1]

[1]Department of Electrical and Electronic Engineering, Imperial College London, London, SW7 2AZ, United Kingdom
[*]irene.garcia-lopez15@imperial.ac.uk

Table of contents:

- **Figure 1**: Recordings extracts of 10s duration showing neck JVP and finger arterial PPG signals for all subjects (n=20).
- **Table 1**: Average time differences between characteristic peaks and waves from neck JVP, finger PPG, and ECG signals.

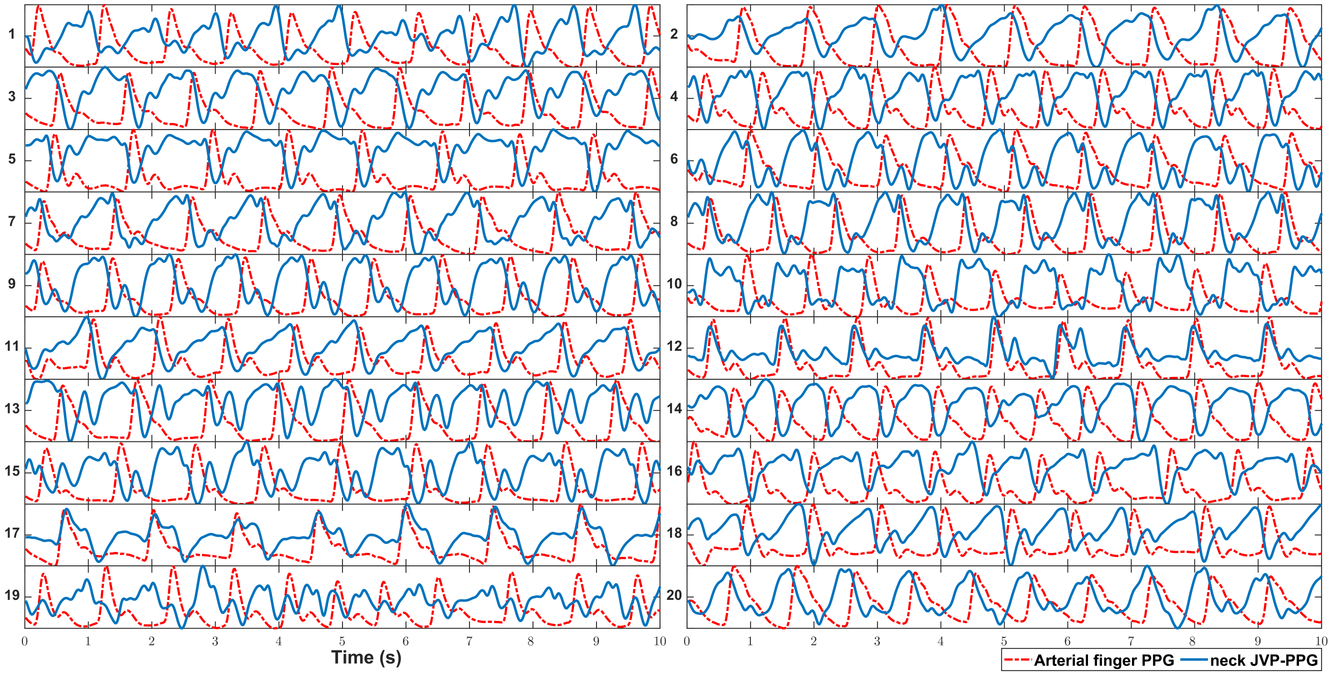

Figure 1. Recordings extracts of 10s duration showing neck JVP and finger arterial PPG signals for all subjects (n=20). Signals were normalized in amplitude in the range [0,1] for visualization purposes.

Table 1. Average time differences between characteristic peaks and waves from neck JVP, finger PPG, and ECG signals. Mean differences (in ms) normalized by the *v-v* interval (1s) are presented for each subject (n=20) together with the standard deviation ($\mu \pm \sigma$). **S** represents the arterial finger PPG systolic peak, **R** is the QRS complex peak of the ECG and **a**,**c**,**v** correspond to the JVP waves. **O** indicates the onset of the JVP pulse.

Subj.	*1*	*2*	*3*	*4*	*5*	*6*	*7*	*8*	*9*
**S-R**	427.6 ± 18.9	380.1 ± 10.5	382.7 ± 54.7	457.3 ± 10.9	355.1 ± 10.8	370.2 ± 15.1	356.1 ± 21.4	387.3 ± 14.4	357.4 ± 8.6
**S-c**	270.8 ± 18.2	205.6 ± 22	248.4 ± 39.6	349.3 ± 13	249.2 ± 16	243.6 ± 18.9	221.1 ± 26	285.2 ± 34.5	285.1 ± 15.6
**c-R**	156.9 ± 5.5	174.5 ± 19.7	134.3 ± 17.4	108 ± 5.2	105.9 ± 5.7	126.6 ± 6.8	135 ± 11.6	102.1 ± 34	72.3 ± 13.4
**c-v**	650.4 ± 49.5	440.8 ± 31	456.2 ± 66.8	435.1 ± 16.7	552.4 ± 25.7	508.7 ± 11.2	518.1 ± 30.5	446.3 ± 40.2	526.1 ± 30.3
**c-a**	215.8 ± 13	222.8 ± 9	231 ± 38.1	121.5 ± 14.3	294.8 ± 36.2	150 ± 14.1	280 ± 34.2	124.2 ± 13.4	127 ± 15.4
**a-v**	434.6 ± 52.7	218 ± 29.6	225.1 ± 42.5	313.6 ± 9.9	257.6 ± 19.1	358.8 ± 12.2	238.1 ± 10.4	322.1 ± 28.9	399.2 ± 16.6
**R-v**	493.5 ± 48.5	266.3 ± 37.2	321.9 ± 81.5	327 ± 17.6	446.4 ± 21.1	382.1 ± 12.5	383.1 ± 36.4	344.2 ± 19.1	453.8 ± 29.8
**R-O**	636.3 ± 22.1	439.3 ± 15.6	483.8 ± 66.3	510.3 ± 7.9	625 ± 18.2	531.8 ± 5.3	515.2 ± 25.6	462.7 ± 9.2	589 ± 15.2
**R-a**	58.9 ± 13.6	48.3 ± 28.1	96.7 ± 48.1	13.5 ± 16.8	188.9 ± 31.8	23.3 ± 12.1	145 ± 39.4	22.1 ± 24.7	54.7 ± 16.1

Subj.	*10*	*11*	*13*	*14*	*15*	*16*	*18*	*19*	*20*
**S-R**	396 ± 32.6	362.6 ± 10.9	368.9 ± 7.9	417.5 ± 27.1	353.7 ± 14.9	374.7 ± 37.3	370.7 ± 43.4	409.5 ± 44.5	396.6 ± 17.3
**S-c**	267.8 ± 8.9	252.6 ± 6.8	191.2 ± 9.9	280.7 ± 18.3	240.2 ± 19.1	263.3 ± 28.1	197.6 ± 9.1	331.3 ± 42	259.3 ± 21.1
**c-R**	128.2 ± 27.7	110 ± 7.8	177.7 ± 15.4	136.8 ± 18.1	113.5 ± 4.7	111.4 ± 12.2	173.2 ± 42.5	78.2 ± 14	137.4 ± 24.3
**c-V**	504.9 ± 34.3	447.4 ± 13.5	500.3 ± 23.6	478.7 ± 15	555.2 ± 38.8	619.3 ± 42.9	590.8 ± 43.2	380.9 ± 58.4	454.3 ± 30.6
**c-a**	251.2 ± 23.3	210 ± 12.2	207 ± 42	222.2 ± 27.1	192.1 ± 37.3	210.2 ± 36.6	231.4 ± 44.1	149.6 ± 19.8	-
**a-v**	253.7 ± 55.3	237.4 ± 11	293.3 ± 28.3	256.5 ± 16.2	363 ± 5	409.1 ± 74.8	359.4 ± 49.1	231.3 ± 63	-
**R-v**	376.7 ± 24.2	337.4 ± 14.9	322.6 ± 11.4	341.9 ± 21	441.7 ± 41.5	507.9 ± 51	417.6 ± 51.4	302.7 ± 59.9	316.9 ± 23.5
**R-O**	562.7 ± 31.3	507.4 ± 10.5	516.3 ± 6.4	570.4 ± 28.7	578.4 ± 21	699 ± 40.7	634 ± 40.8	456.3 ± 67.7	418 ± 5.9
**R-a**	123 ± 46.3	100 ± 13	29.3 ± 36.2	85.4 ± 30.9	78.6 ± 39.9	98.8 ± 26	58.2 ± 8.3	71.4 ± 8.6	-
